# Development of a Multiplex Fluorescence in Situ Hybridization Assay to Identify Coinfections in Young‐of‐the‐Year Smallmouth Bass

**DOI:** 10.1002/aah.10144

**Published:** 2021-10-08

**Authors:** Heather L. Walsh, Vicki S. Blazer, Patricia M. Mazik

**Affiliations:** ^1^ U.S. Geological Survey, Eastern Ecological Science Center at the Leetown Research Laboratory 11649 Leetown Road Kearneysville West Virginia 25430 USA; ^2^ U.S. Geological Survey West Virginia Cooperative Fish and Wildlife Research Unit West Virginia University, Davis College of Agriculture, Natural Resources and Design 313C Percival Hall Morgantown West Virginia 26506 USA

## Abstract

Histopathological assessments of young‐of‐the‐year (age‐0) Smallmouth Bass *Micropterus dolomieu* in the Susquehanna River drainage identified a high prevalence of the myxozoan *Myxobolus inornatus*. This myxozoan infects the connective tissue of the muscle below the skin but is sometimes observed in the esophagus and buccal cavity. In some instances, shallow infections cause breaks in the skin, which could increase the chance of opportunistic bacterial infections. Several microbial pathogens, including *Flavobacterium columnare*, *Aeromonas* spp., and Largemouth Bass virus, have also been cultured from clinically diseased young of year. A multiplex fluorescence in situ hybridization (FISH) assay was developed to determine potential colocalization of *M. inornatus*, *Flavobacterium* spp., and *Aeromonas* spp. infections. With FISH, 75% of age‐0 Smallmouth Bass exhibited *M. inornatus* infections, 10% had *Aeromonas* spp. infections, and 5% had *Flavobacterium* spp. infections, while 3% had coinfections with both bacterial species and *M. inornatus*. The results of the multiplex FISH assay revealed a low occurrence of coinfections of *Flavobacterium* spp. and/or *Aeromonas* spp. with *M. inornatus* in randomly sampled individuals.

Health assessments of young‐of‐the‐year (age‐0) Smallmouth Bass *Micropterus dolomieu* in the Susquehanna River drainage in Pennsylvania have been ongoing for over a decade. Observations of age‐0 Smallmouth Bass mortalities were first documented in the summer of 2005 and have varied in prevalence and spatially and temporally in years since (Walsh et al. 2018). The magnitude of reoccurring disease in age‐0 Smallmouth Bass has been observed at the population level, with a significant decrease in age‐1 Smallmouth Bass and a shift to larger and older fish (Smith et al. 2015). Initial observations from randomly sampled and/or moribund fish with visible skin lesions included the myxozoan parasite *Myxobolus inornatus* (Walsh et al. 2012a) as well as *Flavobacterium columnare* (Chaplin et al. 2009). Subsequently, Largemouth Bass virus (LMBV), other parasites, and multiple *Aeromonas* spp. were identified (Starliper et al. 2013; Smith et al. 2015; Boonthai et al. 2018; Walsh et al. 2018). Abiotic factors such as water quality, contaminants of emerging concern, and periods of increased water temperature may also be associated with disease, but it is likely the cumulative effects of these stressors that present the greatest risk.

One of the most frequently observed parasites infecting age‐0 Smallmouth Bass in the Susquehanna River is *M*. *inornatus*, which infects the connective tissue of the muscle. Plasmodium are often found below the dermis of the skin and can become large enough to rupture the epidermis (Walsh et al. 2012a). Previous studies have shown that primary infections with parasites that cause skin damage increase the likelihood of subsequent infections with pathogenic bacteria (Esch et al. 1976; Roon et al. 2015). For example, Rainbow Trout *Oncorhynchus mykiss* experimentally infected with the ectoparasite *Argulus coregoni* showed susceptibility to increased infections and mortality when exposed to *F. columnare* (Bandilla et al. 2006).

The increased risk of coinfections associated with *M. inornatus* has been a concern, and one method that can be used to visualize coinfections is fluorescence in situ hybridization (FISH). This method has been used in many teleost studies for pathogen detection and identification (Morris et al. 1999; Holzer et al. 2003; Grésoviac et al. 2007; McCarthy et al. 2008; Strepparava et al. 2012) by visualizing the DNA or RNA of a target within a tissue section (Cassidy and Jones 2014). It unifies microbiology, molecular biology, and pathology to answer diagnostic questions that cannot be determined with any one field alone (Frickmann et al. 2017). The objective of this study was to develop a multiplex FISH assay to determine whether age‐0 Smallmouth Bass infected with *M. inornatus* have observable coinfections with *Flavobacterium* spp. and/or *Aeromonas* spp. Age‐0 Smallmouth Bass were sampled from multiple sites in the Susquehanna River drainage in Pennsylvania and histologically examined for the presence of *M*. *inornatus*. Samples were selected based on these findings and used to determine the validity of the multiplex FISH assay.

## METHODS

### Fish sampling and histology

**FIGURE 1:**
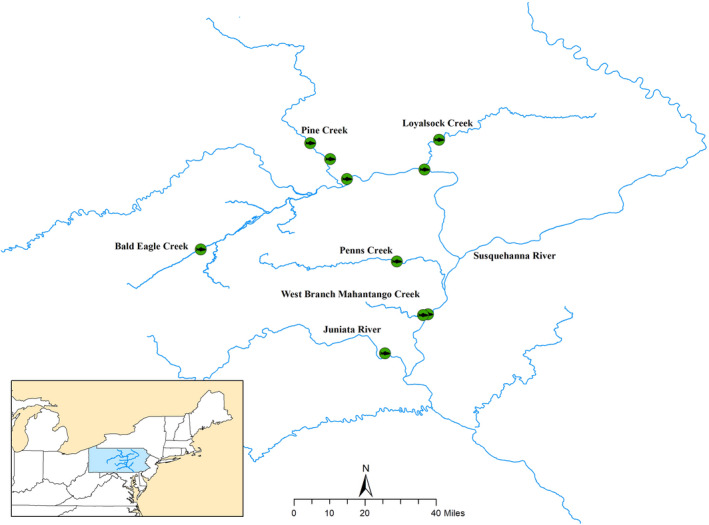
Collection sites (green circles) for young‐of‐the‐year Smallmouth Bass within the Susquehanna River drainage in 2014.

In 2014, age‐0 Smallmouth Bass were sampled from multiple sites in the Susquehanna River drainage with backpack electroshocking equipment (Figure 1). These were random samples that included individuals with and without visible lesions. Fish were euthanized with a lethal dose (350 mg/L) of tricaine methanesulfonate (MS‐222; Syndel USA, Ferndale, Washington), as approved by the Eastern Ecological Science Center’s Institutional Animal Care and Use Committee. An incision was made to open the abdominal cavity, and whole fish were placed in Z‐Fix (Anatech, Battle Creek, Michigan) or PAXgene (PreAnalytiX, Switzerland). Whole bodies were decalcified with EDTA and routinely processed for microscopic examination (Luna 1992). Two longitudinal sections of the head and up to six dorsal, transverse sections of the body were placed in cassettes, dehydrated in graded concentrations of alcohol, embedded into paraffin, sectioned at 5 μm, and stained with hematoxylin and eosin.

### Fluorescence in situ hybridization

Paraffin sections were cut at 5 µm and placed on positively charged slides (Fisherbrand Superfrost Plus Microscope Slides; Fisher Scientific, Waltham, Massachusetts) coated with poly‐L‐lysine. Sections were deparaffinized with three changes of Pro‐Par (Anatech, Battle Creek, Michigan) for 5 min each and rehydrated with a graded ethanol series consisting of 100, 95, 80, 70, and 50% for 3 min each and rinsed in phosphate‐buffered saline (PBS) three times for 1 min each. Incubation in 0.2N HCl was conducted for 20 min at room temperature as an added protein extraction and target hydrolysis step and was followed by a rinse in PBS for 1 min. Tissues were permeabilized in Proteinase K (Sigma‐Aldrich, St. Louis, Missouri) at a concentration of 100 µg/mL in PBS for 10 min at 37°C; however, this step was excluded for PAXgene‐fixed tissues. Following the Proteinase K incubation, sections were rinsed twice in 2% glycine in PBS for 3 min each and rinsed in PBS for 5 min. A hybridization solution consisting of 50% deionized Formamide, 20% 4× standard saline citrate (SSC; 20× SSC = 3.00 M NaCl, 0.34 M sodium citrate, pH 7.0), 5% heat‐denatured single‐strand salmonid sperm DNA (at 10 mg/mL; Sigma‐Aldrich), 20% dextran sulfate (50%, volume per volume), and 2% 50× Denhardt’s solution was added to each slide with a probe concentration of 10 ng/µL, and a Hybriwell coverslip (Grace Bio‐Labs, Bend, Oregon) was applied. Control slides were covered with hybridization solution without probes. Probe‐positive slides and controls were denatured at 95°C for 10 min and then placed in an InSlide Out hybridization oven (Boekel, Feasterville, Pennsylvania) overnight at 40°C. The following day, the slides were washed in 4× SSC for 10 min at room temperature, twice in 2× SSC for 10 min at 37°C, and twice in 0.5× SSC for 5 min at room temperature. After a wash in PBS for 10 min at room temperature, slides were counterstained in Sudan Black B (1% in 70% ethyl alcohol) for 5 min to reduce autofluorescence. Following staining, slides were rinsed three times with PBS for 3 min each, and a glass coverslip was mounted with ProLong Diamond Antifade Mountant (Life Technologies, Eugene, Oregon).

Conserved 16S rRNA gene sequences of *Aeromonas* spp. and *Flavobacterium* spp. (Table 1) were used for probe development and purchased from IDT DNA (San Diego, California) with AlexaFluor tags on the 5′ end. The probe for *M. inornatus* was originally species‐specific (Walsh et al. 2012a); however, a recent addition to GenBank of the myxozoan *Myxobolus doubleae* (Milanin et al. 2020) revealed the potential for cross‐reactivity. The sequences of the two species (accession numbers MK592013.1 and JN896706.1) were aligned in Geneious 10.1.3 with the ClustalW alignment tool (default settings) and revealed a 95% identity match. However, while they are closely related, *M*. *doubleae* was identified on the West Coast of the United States, infects a different host (Yellow Perch *Perca flavescens*) and infection site (gill filament), and is slightly different in size (Milanin et al. 2020). Positive controls of fish infected with *M. inornatus*, *F*. *columnare*, *A. hyrophila*, and *A. sobria* were also included. The probe developed for *M. inornatus* was tested for specificity using age‐0 Smallmouth Bass infected with *M. inornatus* and Smallmouth Bass infected with two other myxozoans, *M. branchiarum* (Walsh et al. 2012b) and a currently undescribed myxozoan that infects the kidney. Positive controls for the *Flavobacterium* spp. probe consisted of tissue samples from juvenile Channel Catfish *Ictalurus punctatus* (spleen, liver, head kidney, and skin) immersed in a *F. columnare* bath and were provided by the U.S. Department of Agriculture Aquatic Animal Health Research Laboratory (Auburn, Alabama). Tissue samples of spleen and kidney from Goldfish *Carassius auratus* with a primary cyprinid herpesvirus 2 infection colonized by *A*. *hydrophila* and skin of koi (domesticated version of Common Carp *Cyprinus carpio*) with multiple lesions infected with *A. sobria* were provided by the Department of Pathobiological Sciences at Louisiana State University School of Veterinary Medicine (Baton Rouge, Louisiana) and served as positive controls for the *Aeromonas* spp. probe.

**TABLE 1:** 16S rRNA gene sequences of in situ hybridization probes used to detect *Aeromonas* spp., *Flavobacterium* spp., and *Myxobolus inornatus* in young‐of‐the‐year Smallmouth Bass.

Probe	Sequence	Original publication
*Myxobolus inornatus*	5'‐/5Alex546N/TCG ACG CCC TCC CTG ACT CG‐3'	Walsh et al. 2012
*Flavobacterium* spp.	5'‐/5Alex488N/ACC CCT ACC CAT CGT C‐3'	Strepparava et al. 2012
*Aeromonas* spp.	5'‐/5Alex546N/CTA CTT TCC CGC TGC CGC‐3'	Kämpfer et al. 1996

## RESULTS

### Fish Sampling and Histopathology

**FIGURE 2:**
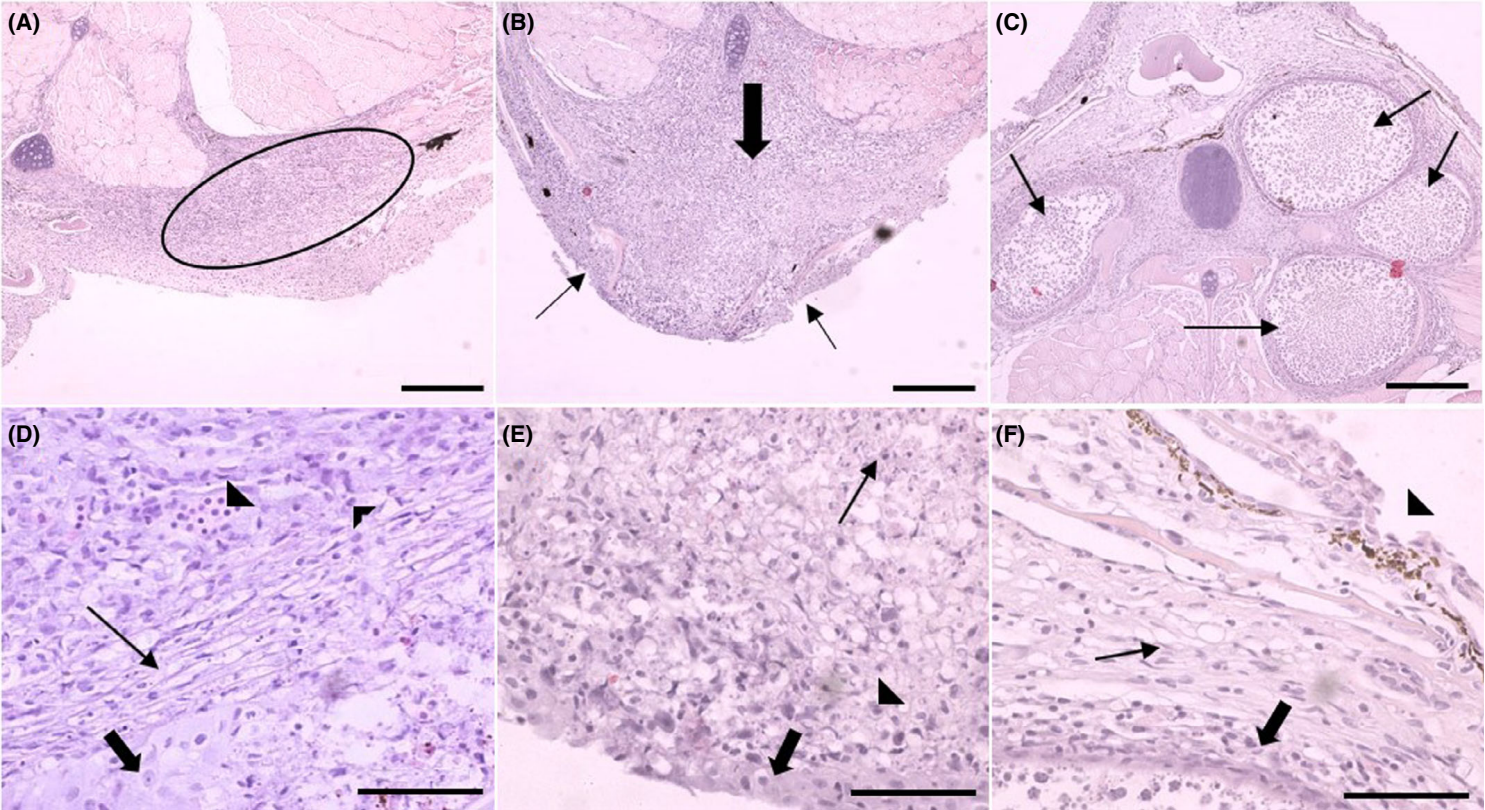
Cross sections of young‐of‐the‐year Smallmouth Bass (hematoxylin and eosin stain) chosen to analyze with FISH, showing **(A)** an area of inflammation (oval) that may be associated with *M. inornatus* and **(D)** a higher magnification depicting a vessel with red blood cells (solid arrowhead), small mononuclear cells (thin arrowhead), vacuolated fibroblasts in dermis (thin arrow), and hypertrophied epithelial cells (thick arrow) next to an area of necrosis. Panel **(B)** shows a skin lesion with inflammation (thick arrow) spreading into the subepidermal layers and surrounding muscle and focal areas of erosion (thin arrows), and panel **(E)** shows a higher magnification depicting necrosis (thin arrow), hypertrophied epithelial cells (thick arrow), and small mononuclear cells (arrowhead). Panel **(C)** shows multiple *Myxobolus inornatus* plasmodia (thin arrows), with **(F)** a higher magnification depicting an erosion of the epidermis (arrowhead), vacuolated fibroblasts in the dermis (thin arrow), and small mononuclear cells (thick arrow) surrounding the plasmodium. The scale bar = 20 μm for panels (A), (B), and (C); the scale bar = 5 μm for panels (D), (E), and (F).

**FIGURE 3:**
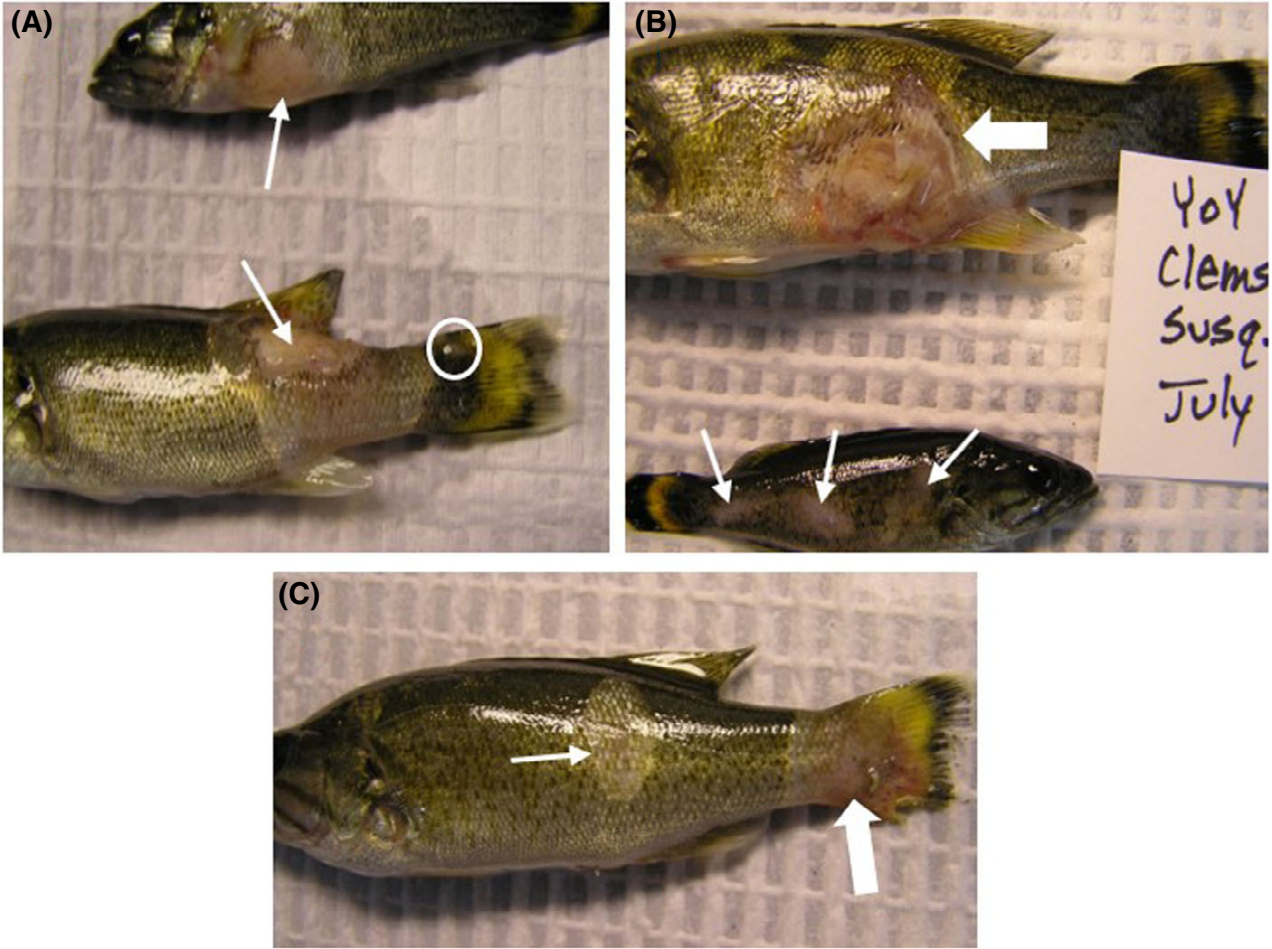
Various types of gross lesions observed on young‐of‐the‐year Smallmouth Bass in the Susquehanna River drainage. Panel **(A)** shows spreading skin erosions with necrotic ulceration exposing the underlying dermis (arrows) and a pale white cyst at the base of the caudal fin (circle) caused by *M. inornatus*. In panel **(B)**, the top fish displays a more advanced spreading skin ulcer with epidermal, dermal, and muscle necrosis and degeneration and hemorrhaging (bold arrow) and the bottom fish displays focal areas of erosion (thin arrows). Panel **(C)** shows an early stage spreading lesion (thin arrow) and a reddened area of erosion at the base of caudal fin (bold arrow).

A total of 479 age‐0 Smallmouth Bass (approximately 20 fish per site) were sampled in 2014 from 20 sites within the Susquehanna River drainage. With histopathology, 173 (173/479; 36%) age‐0 Smallmouth Bass had *M. inornatus* infections in various areas of the body, including the connective tissue below the dorsal and caudal fins, deep in the connective tissue of the muscle, and in areas of the operculum. Ninety‐six fish were used for FISH and were selected if *M. inornatus* infections were uncertain with histopathology (only inflammation was observed; Figure 2A, D), if there was a skin lesion (Figure 2B, E), or if there were heavy or severe *M*. *inornatus* infections (Figure 2C, F). Histologically, skin lesions included the presence of epidermal inflammation and necrosis, sometimes spreading into subepidermal tissue (dermis and muscle) with inflammation dominated by small mononuclear cells. In some instances, these lesions were observed penetrating the muscle. Grossly, lesions varied in appearance and included focal raised areas (Figure 3A), pale discolored areas of the skin (Figure 3A–C), and erosions (Figure 3C). Within some of the discolored areas, pale and/or reddened ulcerations (Figure 3A, B) were observed.

### Fluorescence in Situ Hybridization

**FIGURE 4:**
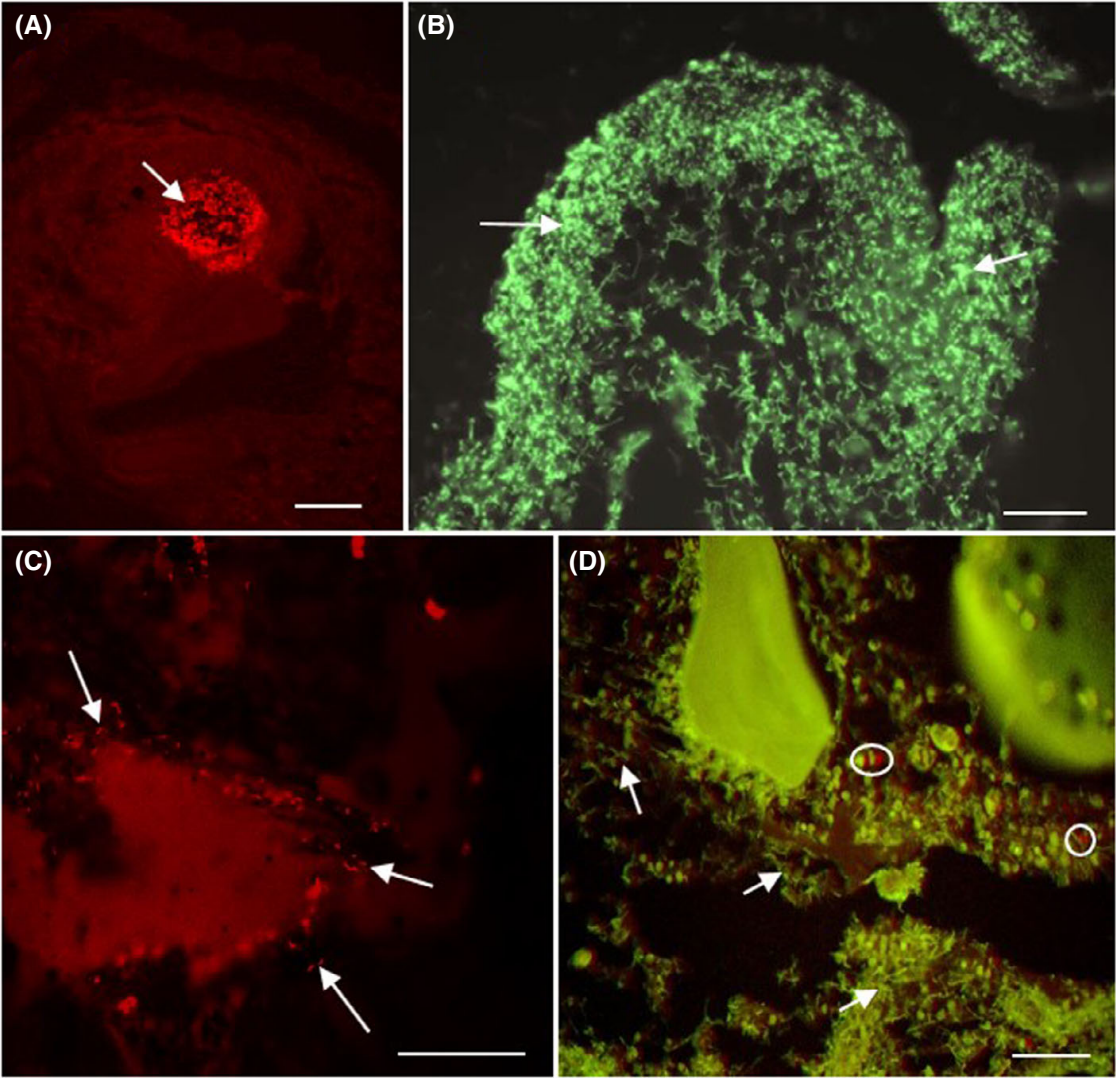
Fluorescence in situ hybridization (FISH) of samples from young‐of‐the‐year Smallmouth Bass tissues, showing **(A)** *Myxobolus inornatus* plasmodium (arrow) infecting the connective tissue of the muscle (scale bar = 50 μm), **(B)** *Flavobacterium* spp. (filamentous bacteria; arrows) in the skin (scale bar = 10 μm), **(C)** *Aeromonas* spp. (rod‐shaped bacteria; arrows) in the gut (scale bar = 5 μm), and **(D)** a coinfection of loose spores of *M. inornatus* (circles) and *Flavobacterium* spp. (filamentous bacteria; arrows) infecting the connective tissue and soft cartilage around the bone in the caudal peduncle (scale bar = 10 μm).

Positive control samples showed that the probes for *Flavobacterium* spp., *Aeromonas* spp., and *M. inornatus* successfully hybridized to their targets. Of the age‐0 Smallmouth Bass analyzed with FISH, 5 exhibited *Flavobacterium* spp. infections (5/96; 5%), 10 exhibited *Aeromonas* spp. infections (10/96; 10%), and 72 (72/96; 75%) exhibited *M*. *inornatus* infections (Figure 4). Coinfections with *M. inornatus* were observed in 4 of the 5 fish with *Flavobacterium* spp. (4/96; 4%; Figure 4D) and 3 of the 10 with *Aeromonas* spp. (3/96; 3%), one fish had a coinfection of *Aeromonas* spp. and *Flavobacterium* spp. without the presence of *M. inornatus* (1/96; 1%), and three fish had coinfections of all three pathogens (3/96; 3.13%). Finally, there were six fish (6/96; 6%) that were positive for *M. inornatus* in areas of inflammation that did not present visible cysts or spores with routine histology.

Location of bacteria varied, with a majority of *Aeromonas* spp. observed in the gut (9/10; 90%) and one incidence of an *Aeromonas* spp. infection in a skin lesion (1/10; 10%). *Aeromonas* spp. were observed in the lumen of the gut, not associated with intestinal lesions, and may have been part of the normal microflora (González et al. 2001). *Flavobacterium* spp. infections were always observed in skin lesions (5/5; 100%) and were not observed in areas of normal skin or gut. Although some species of these two bacterial genera can be found on normal skin (i.e., skin without lesions; González et al. 2001), the lack of findings on normal skin in this study could be due to the relatively low abundance in microflora found on healthy fish skin (Austin 2002).

## DISCUSSION

The importance of coinfections of various pathogens and parasites is increasingly recognized in both wild and cultured fish populations (Kotob et al. 2016; Abdel‐Latif et al. 2020). In fish, the mucosal layer of skin plays an important role in immune function and is the first line of defense against pathogens. However, when this layer is damaged, fish can become vulnerable to subsequent infections (Esteban 2012). In many studies, bacterial infections have been documented secondarily after primary infections with parasites that cause skin erosion (Esch et al. 1976; Pylkkö et al. 2006; Xu et al. 2012, 2014; Zhang et al. 2015). Myxozoans are known to infect fish by invasion of the mucosal layer and skin (Gόmez et al. 2014) and can create portals for secondary infections. Although coinfections were observed in this study, bacterial infections were not always directly associated with *M. inornatus*. Both *Aeromonas* spp. (such as *A. hydrophila* and *A*. *sobria*) and *Flavobacterium* spp. (such as *F. columnare*) are capable of infecting fish externally and internally (Camus et al. 1998; Loch and Faisal 2015) and have also been found in the intestinal microflora of normal, healthy juvenile fish (Ringø and Birkbeck 1999).

The FISH assay successfully identified early stages of *M. inornatus* in areas where only inflammation was observed with histopathology. Other studies have utilized FISH when histopathology alone was insufficient, such as for the detection of early life stages of *Parvicapsula pseudobranchicola* (Markussen et al. 2015) or to understand how various myxozoans migrate through the host (Morris et al. 1999; Holzer et al. 2003; Bjork and Bartholomew 2010). Studies have also utilized FISH for the detection of parasites that are associated with nonspecific signs (Frickmann et al. 2012) or when infectious agents require rapid identification or are difficult to grow with culture techniques (Frickmann et al. 2017). In this study, the advantage of FISH over culturing was the visualization of where bacterial infections occurred and whether they co‐occurred with *M. inornatus*. Bacterial cultures alone would have missed the detection of bacteria subdermally and within internal tissues. For example, the *Aeromonas* spp. identified with FISH were primarily located within the lumen of the gut, an area where bacteria are not routinely cultured. The FISH assay also allowed a direct association to be made between *M*. *inornatus* and bacteria‐positive samples and histopathological alterations. Methods such as PCR or quantitative PCR are useful for providing prevalence data but are prone to contamination and do not offer the ability to visualize targets that coexist.

There are many species of myxozoan parasites that are primary pathogens of fish (Lom and Dyková 2006), and they are often more pathogenic to juvenile fish (Ryce et al. 2005; Foott et al. 2007; Bolick et al. 2013; Ahmad and Kaur 2017) as their adaptive immune response is still underdeveloped (Zapata et al. 2006; Uribe et al. 2011). Additionally, the effects of myxozoan infections can be exacerbated by other factors. The myxozoan *M*. *cerebralis*, for example, is associated with mortality in fingerling Rainbow Trout, and mortality may increase by exposure to high water temperatures (Schisler et al. 2000). The pathogenic effects of myxozoans vary and are dependent upon environmental factors (such as temperature and water quality), host immunocompetence, the species of myxozoan, and the target organ of infection (Gόmez et al. 2014). Age‐0 Smallmouth Bass in the Susquehanna River drainage are periodically exposed to low dissolved oxygen and elevated temperatures, especially in the summer during low flow (Chaplin et al. 2009). Additionally, complex mixtures of surface water contaminants (Reif et al. 2012; McClure et al. 2020) and tissue contaminants (Walsh et al. 2018) have been documented. Thus, it is possible that environmental conditions could exacerbate the effects of *M. inornatus*, resulting in a more primary role in mortality in age‐0 Smallmouth Bass with or without secondary infections. This is something that should be taken into consideration for future monitoring and population surveys.

To conclude, this study was successful at the development of a multiplex FISH assay to identify coinfections of pathogens in wild, age‐0 Smallmouth Bass. The FISH results showed that infections of *Aeromonas* spp. and *Flavobacterium* spp. can be associated with *M. inornatus*. However, the high prevalence of *M*. *inornatus* infections suggests that this may be a primary pathogen of age‐0 Smallmouth Bass, but further research will be needed to determine if it is associated with immunosuppression or primes the host for secondary infections (Kotob et al. 2016). The FISH assay showed that the frequency of *Aeromonas* spp. and *Flavobacterium* spp. was low in this study. In future studies, other microbial pathogens, such as LMBV, should also be considered in addition to PCR to compare prevalence and establish a relative detection limit of FISH. In laboratory exposures with LMBV, age‐0 Smallmouth Bass developed skin lesions (Boonthai et al. 2018) similar to lesions observed on wild age‐0 Smallmouth Bass from the Susquehanna River drainage. Additional studies are required to better understand the role of environmental stressors on coinfections of the various organisms observed and cultured, as well as the pathogenicity of *M. inornatus*, the role it plays in mortality of age‐0 Smallmouth Bass, and the environmental factors associated with proliferation of the parasite and resistance of the host in order to mitigate the effects of age‐0 Smallmouth Bass disease.
